# Advancing perinatal patient safety through application of safety science principles using health IT

**DOI:** 10.1186/s12911-017-0572-8

**Published:** 2017-12-19

**Authors:** Jennifer Webb, Asta Sorensen, Samantha Sommerness, Beth Lasater, Kamila Mistry, Leila Kahwati

**Affiliations:** 1 RTI International, 3040 E. Cornwallis Rd, Research Triangle Park, NC 27709 USA; 2 University of Minnesota School of Nursing, 5-140 Weaver-Densford Hall, 308 Harvard Street SE, Minneapolis, MN 55455 USA; 3 Agency for Healthcare Research and Quality, 5600 Fishers Lane, Mailbox: 06E81B, Rockville, MD 20857 USA

**Keywords:** Perinatal health, Labor and delivery, Health IT, Electronic health record (EHR), Patient safety, Quality improvement, Safety science principles, Standardization, HIT implementation

## Abstract

**Background:**

The use of health information technology (IT) has been shown to promote patient safety in Labor and Delivery (L&D) units. The use of health IT to apply safety science principles (e.g., standardization) to L&D unit processes may further advance perinatal safety.

**Methods:**

Semi-structured interviews were conducted with L&D units participating in the Agency for Healthcare Research and Quality’s (AHRQ’s) Safety Program for Perinatal Care (SPPC) to assess units’ experience with program implementation. Analysis of interview transcripts was used to characterize the process and experience of using health IT for applying safety science principles to L&D unit processes.

**Results:**

Forty-six L&D units from 10 states completed participation in SPPC program implementation; thirty-two (70%) reported the use of health IT as an enabling strategy for their local implementation. Health IT was used to improve standardization of processes, use of independent checks, and to facilitate learning from defects. L&D units standardized care processes through use of electronic health record (EHR)-based order sets and use of smart pumps and other technology to improve medication safety. Units also standardized EHR documentation, particularly related to electronic fetal monitoring (EFM) and shoulder dystocia. Cognitive aids and tools were integrated into EHR and care workflows to create independent checks such as checklists, risk assessments, and communication handoff tools. Units also used data from EHRs to monitor processes of care to learn from defects. Units experienced several challenges incorporating health IT, including obtaining organization approval, working with their busy IT departments, and retrieving standardized data from health IT systems.

**Conclusions:**

Use of health IT played an integral part in the planning and implementation of SPPC for participating L&D units. Use of health IT is an encouraging approach for incorporating safety science principles into care to improve perinatal safety and should be incorporated into materials to facilitate the implementation of perinatal safety initiatives.

## Background

The use of health information technology (IT) can be an important strategy for improving patient safety [1]. Health IT applications are increasingly used within Labor and Delivery (L&D) units to promote safety including clinical decision support systems, computerized medication order entry, and medication safety devices [2]. These health IT applications aid providers in treatment decisions, support more complete and timely documentation, promote medication safety, and enable standardized data collection for quality improvement [2–6].

Health IT applications can be used as a means for applying safety science principles to quality and safety initiatives as in the Agency for Healthcare Research and Quality’s (AHRQ) Comprehensive Unit-based Safety Program (CUSP), which we recently adapted for use in L&D Units as the AHRQ Safety Program for Perinatal Care (SPPC) [7]. CUSP aims to improve the unit culture of safety by combining teamwork and communication, leadership, and safety science principles [8]. These principles include standardizing processes, creating independent checks, and learning from defects. Health IT applications such as computerized provider order entry (CPOE), clinical decision support systems, and “smart” infusion pumps can aid in standardizing processes on L&D units. Standardizing nomenclature and data elements to be incorporated into electronic health records (EHR) for documenting procedures and patient status not only helps to improve real-time communication but allows for EHR documentation to be collected, reported, and compared across different units and organizations [9, 10]. Electronic checklists and other cognitive aid tools can be integrated and used in EHRs to create independent checks for both common L&D procedures (e.g., cesarean section) and obstetric emergencies such as shoulder dystocia and hemorrhage among others. Incorporating checklists and tools into EHR systems can guide providers through clinical processes; this can reduce cognitive burden and help to ensure no important steps are forgotten. [4, 10]. The safety science principle of learning from defects requires data about what happened surrounding an untoward event which can be captured and retrieved from checklists and other standardized documentation tools for evaluation of defects and quality improvement activities [4, 5].

Several studies have shown improvements in L&D unit safety through the use of health IT applications that enable the safety science principles. Provost & Gray found improved physician compliance with documentation of indication for elective labor induction after implementing a decision support system when compared to before implementation [6]. Deering et al. found that documentation of several critical elements associated with shoulder dystocia care was significantly improved following the implementation of an electronic standard checklist. Important care components (e.g., gentle traction, McRoberts maneuver) and documentation elements (e.g., head-to-body delivery interval and presence/absence of clavicular fracture) were all found to be documented more frequently with use of the checklist [5]. Eden et al. found that implementation of an obstetric-specific EHR resulted in more complete documentation in records when compared with paper-based records [11].

Although several examples of successful health IT applications for improving patient safety have been published, information on how to leverage health IT to enable the safety science principles is often lacking in perinatal safety initiatives. As the role of health IT applications in perinatal settings is continually expanding, their use as a strategy for operationalizing safety science principles needs further evaluation. This paper describes how L&D units that implemented the AHRQ Safety Program for Perinatal Care (SPPC), a comprehensive perinatal safety program based on CUSP, incorporated the use of health IT to enable safety science principles for improving the culture of patient safety, teamwork and communication, and the quality of care processes [7].

## Methods

### Design of the Safety Program for Perinatal Care

The AHRQ SPPC was designed to facilitate coordination and teamwork in the L&D unit to improve patient safety and quality of care and decrease maternal and neonatal adverse events. The program consists of three program pillars: (1) fostering a culture of teamwork and communication, (2) implementing perinatal safety strategies, and (3) establishing a program of in situ simulations. Perinatal safety strategies were designed to apply the safety science principles from CUSP (i.e., standardizing, creating independent checks, learning from defects) to common obstetric processes (e.g., EFM, medication administration, cesarean section) and obstetric emergencies (e.g., obstetric hemorrhage, shoulder dystocia, and rapid response systems) [8]. Participating L&D units received a series of web-based trainings, technical assistance, and data feedback reports to aid in the implementation of the program. Units were allowed to select and customize the perinatal safety strategies that best met their local culture and needs. Table 1 lists the number of units that implemented each of these strategies during the implementation phase.

**Table 1:** Perinatal safety strategies selected by L&D units in the SPPC, 2015–2016

Strategy	Number of L&D units^a^
Safe electronic fetal monitoring	21
Rapid response systems	25
Safe medication administration: oxytocin	22
Safe medication administration: magnesium sulfate	9
Safe practices related to cord prolapse	7
Safe practices related to shoulder dystocia	18
Safe practices related to obstetric hemorrhage	32
Safe cesarean section	13

^a^The number of units reporting the selection of this strategy at final data collection

### SPPC participants

Forty-six L&D units completed participation in SPPC implementation between December 2014 and January 2016. Hospital and unit characteristics are summarized in Table 2.

**Table 2:** Characteristics of the 46 L&D units that completed participation in the AHRQ SPPC, 2015–2016

Hospital or L&D unit characteristic	Frequency (%) or median (IQR)
Publicly owned	9 (20%)
Rural referral center	5 (11%)
Median number of hospital beds	9 9(IQR 6 to 14)
Level 1 Basic neonatal care	14 (30%)
Level 2 Specialty neonatal care	12 (26%)
Level 3 Subspecialty neonatal care^a^	20 (44%)
Graduate medical program in obstetrics and gynecology	12 (26%)
Median annual number of births	1430 (IQR 513 to 2692)
L&D patient age^b^	27 (IQR 26 to 29)
Proportion of L&D patients that are white^c^	68 (IQR 48% to 85%)

^a^Includes Level 3A, 3B, 3C, and 3D

^b^Each unit reported the mean age of L&D patients treated on their unit. The value reported here represents the median of the ages reported by units

^c^Each unit reported the racial and ethnic composition of L&D patients treated on their unit. The value reported here represents the median of the proportion of white patients reported by units

### Evaluation methods

Participating sites submitted hospital and unit characteristics at baseline and following program implementation, semi-structured interviews were conducted with representatives of 46 of the participating L&D units. Table 3 summarizes the different types of organizational roles that participated in the interviews. Interviews were conducted by phone and in-person and lasted 1 h. Most interviews were conducted individually, in dyads, or in triads; a few were conducted in a focus group format.

**Table 3:** Number of interviewees by organizational roles

Organizational role	Number interviewed
Nursing Directors	18
Nursing (e.g., Nurse Mangers, Perinatal Clinical Nursing Specialists, Staff Nurses, Educators)	61
Physician (e.g., OB/GYN, Anesthesiologist)	10
Senior Leadership (Hospital President, VP of Nursing, Chief Clinical Officer)	7
Administrator (e.g., Patient Care Administrator for OB, Perinatal Administrator)	5
Quality (e.g., Quality Director, Quality Coordinator)	12
Data Specialist (Data Coordinator, Data Manager)	9
Other (e.g., Project Manager, Patient Safety Officer, Informatics Coordinator)	9
Total Interviewed	131

The interviews focused on unit staff experiences with program implementation and were based on domains from the Consolidated Framework for Implementation Research (CFIR) [12]. Interview protocols were customized and focused on perinatal safety strategies implemented by the unit. Interviews focused on implementation team structure and roles, experience with implementation and use of TeamSTEPPS® teamwork and communication techniques, the unit’s selected perinatal safety strategies, and use of in situ simulations [13]. Additional questions explored the use of the EHR in program implementation, experiences with collecting and reporting perinatal safety data, observed impacts of the program, and plans for sustainability.

Interviews were audio-recorded with permission of the interviewees and transcribed verbatim. NVivo version 10 (QSR International) was used to import and analyze all qualitative data. To capture emerging themes within and across the L&D units, we used deductive and inductive analysis techniques. A codebook based on select CFIR constructs was used for deductive analysis. To achieve consistency in data coding and interpretation, two analysts coded the first three interviews independently and assessed inter-rater reliability (percent agreement 95.5%, K = 0.58) to identify and resolve inconsistencies in application of the codebook. The remaining interviews were coded by individual coders. Coded data were further analyzed using inductive analysis techniques to identify emerging themes in the data. The evaluation was not considered to be human subjects research by the RTI International Institutional Review Board.

## Results

Thirty-two L&D units (70%) incorporated EHR or other health IT applications during implementation of the SPPC. Two additional units developed plans for modifying their EHRs but did not receive approval from their organizational leadership to implement the modifications. When comparing L&D units that incorporated health IT with the units that did not, few differences were found related to hospital and unit characteristics between the two groups; total births (use of health IT vs. non-use of health IT, 2371 vs. 1353), number of labor and delivery beds of the hospital (11.1 vs. 7.8), age of mother (27.8 vs 27.4), percent of L&D patients that are white (62.9% vs 71.9%), percent of hospitals that were rural referral center (9.7% vs. 15.4%), percent of hospitals that were publicly owned (22.6% vs. 15.4%), percent of hospitals with a residency program (68.8% vs. 84.6%), percent of hospitals who reported a level 1 NICU basic neonatal care (22.6% vs. 53.9%), level 2 NICU specialty neonatal care (29.0% vs. 23.1%), and level 3 NICU sub-specialty neonatal care (48.4% vs. 26.0%). None of these differences were found to be statistically significant. Participating SPPC hospitals mentioned use of several different EHR systems and products on the unit including EPIC (EHR) and EPIC Stork, Meditech (EHR), GE Centricity™ (EHR) and Centricity Perinatal, Allscripts Sunrise™ (EHR), Clinical Computer Systems OBIX®, and Midas + ™ among others. Units that did incorporate health IT utilized and made modifications to their EHR systems to align with safety science principles for improving the culture of patient safety and quality of care processes on their unit.

### Use of health IT for standardization

Through the use of safety science principles, program participants were encouraged to standardize procedures and processes on their units and a number of them used or modified their EHR systems to apply this principle. Unit staff made changes to standardized care processes through use of their health IT applications by developing new or revising standard EHR-based order sets, developing or standardizing electronic documentation templates, standardizing EFM documentation processes within the EHR, and installing smart pumps and other technology to standardize medication administration. The rest of this section details these findings.

Unit staff planned and executed efforts to develop new or modify existing order sets in their EHRs related to EFM, administration of oxytocin and magnesium sulfate, and obstetric hemorrhage. Nine units developed new or modified order sets to align with other changes made through SPPC to standardize protocols or policies on the unit. Units also reviewed existing order sets and made changes based on provider consensus to ensure standardized orders were being used across the unit. Units involved multidisciplinary teams to plan and develop new order sets including physicians, nurses, pharmacists, IT staff, and leadership committees to ensure that the order sets were based on up-to-date, evidence-based information.

Seven participating units used or modified their EHRs to add improved and standardized methods for documenting procedures and interventions. Units incorporated new data fields, drop-down menus, templates, and pop-up alerts to their EHR systems for documenting certain care processes, such as the management of shoulder dystocia. These modifications helped to ensure that the correct data elements were included by making the documentation more structured, standardized, and easier to record and prevented important data from being left out of the record.

Six units worked to standardize processes and EHR documentation of EFM. Units configured National Institute of Child Health and Human Development (NICHD) nomenclature for EFM interpretation using discrete data fields for classifying fetal status (normal, indeterminate, abnormal) in their EHRs. These improvements made it easier for nurses and providers to document and communicate findings in a standardized way across all clinicians with simple clicks in the EHR. These units developed templates in the EHR that prompted clinicians to provide documentation including further descriptions of the elements for the category of fetal status selected. Use of the newly configured discrete data fields allowed for easily retrievable data to create reports of all abnormal strips documented every month. These reports allowed teams to review the strips, assess the completeness and accuracy of documentation, and appropriateness of the clinical intervention. Regular peer review facilitated continuous feedback in care improvements on the unit. These reports also improved efficiency, as they replaced time consuming manual data retrieval of this data often through reviewing free text note sections in the records.

Other standardization changes related to installation of new smart pumps, which allowed administration of medication using drug dosing libraries programmed on the pump to help prevent dosing errors. A few units changed to single standardized concentrations of oxytocin infusion bags in combination with smart pumps, to further eliminate the need for dosing calculations or reprogramming of the pumps. Four L&D units also standardized medication dispensing through use of hemorrhage medication kits for quicker medication acquisition. Unit staff worked with their pharmacy and IT departments to program these hemorrhage medication kits into their automated medication dispensing stations so that during a hemorrhage emergency unit staff could select the hemorrhage kit, which activates all the necessary medication drawers to open at once so they can quickly obtain all the proper medications. Creating a hemorrhage kit sped up the process and resulted in quicker retrieval of needed medications during an emergency situation and no longer required staff to type in each medication and obtain them individually.

### Use of health IT to support independent checks

L&D units were encouraged to apply the safety science principle of creating independent checks into their care processes and procedures to establish checks and reminders that reinforce perinatal safety. Some SPPC units modified their EHRs to establish these independent checks through use of electronic checklists, electronic hemorrhage risk assessments during admission to L&D, electronic communication and handoff tools, and transitioning to use and documentation of quantifiable blood loss during deliveries and hemorrhage episodes.

Three units configured their EHRs to build in checklists for oxytocin administration and shoulder dystocia management. Checklists, which were promoted as cognitive aids within the SPPC, were helpful in preparing for medication administration or a procedure to ensure that everything was in place and that the safety of the patient was continually monitored throughout the process. To promote regular use of the checklists once implemented, one unit educated staff to use the checklist as a tool to help guide procedures for taking care of their patients in an effort to encourage staff to shift from treating the patient first and charting later.

Eight units created independent checks within their EHRs by adding an electronic hemorrhage risk assessment to be completed upon a patient’s admission to the hospital, to raise awareness about an individuals’ increased risk for hemorrhage in order to be prepared and have resources and equipment available at the time of delivery. When this information is included in the EHR, it is available for all unit staff and ensures that it is not lost during shift changes. Units also made efforts to build risk assessment reports to replace time-consuming manual chart reviews for checking compliance with hemorrhage risk assessments done on the unit.

Unit staff also described creating independent checks on their units through the use of electronic communication tools. Four units used EHR-embedded handoff tools during transitions on the unit, which included required signoffs from both nurses to improve and document communication during shift changes. Two units built tools for SBAR (a communication technique from TeamSTEPPS that refers to summarizing information using four components—Situation, Background, Assessment, Recommendation) into their EHRs to conduct bedside reports during handoffs [13]. These tools ensure patient information is disseminated during shift changes and allows for open discussion between staff. Units also made efforts to document information from debriefings held after significant events occurred on the unit. One unit had a debriefing tool built into its EHR specifically for shoulder dystocia occurrences to document what went well and what changes could be made for future occurrences. These debriefs are designed for open communication among all care team members to create checks and question potential deviations in standardized practices or protocols or other perinatal safety concerns. Using these electronic embedded communication tools helped to ensure that nurses and providers on the unit are documenting events and procedures similarly in their records while also having patient information readily available whenever needed.

Creating independent checks in the EHR regarding the quantification of blood loss (QBL) during deliveries and obstetric hemorrhage events was common among participating L&D units. Quantification of blood loss refers to a formal, accurate, and cumulative assessment of ongoing blood loss using as quantitative methods as possible, such as calibrated drapes and containers, and weighing of blood soaked items. This approach identifies excessive blood loss earlier than approaches that rely on non-quantitative methods as clinicians have been shown to underestimate blood loss [14]. As part of participation in the SPPC, many L&D units decided to transition from estimating blood loss to using QBL and worked with their IT departments to refine QBL care processes and documentation in their EHRs. One unit had its IT staff put all QBL fields on one screen for easier documentation and aid in programming to ensure that all documentation was correlating correctly and totals were calculated accurately. Another unit that transitioned to QBL measurement realized that documenting QBL in its EHR was not being done consistently. To remedy this, the team made changes in its EHR to create hard stops, which prevented staff from being able to close out of a chart until the blood loss was documented in the record. This change has helped to ensure that all pieces of the documentation are accurately entered and correct blood loss totals are measured throughout different phases of labor and delivery. Unit staff noted the importance of buy-in from providers and staff when making these types of changes and used multiple methods for educating providers and staff on these changes including discussing in meetings, sending educational materials to staff to review, and promotion through organization leadership.

### Use of health IT for learning from defects and monitoring processes of care

L&D units participating in SPPC were encouraged to track and report process and clinical outcome measures related to perinatal safety throughout the implementation period to use in discussions to learn from defects and to monitor each unit’s implementation progress. Discussions to learn from defects aids units to analyze near misses and adverse events that occurred on the unit and in developing strategies for ensuring safety improvements are implemented to prevent future occurrences. Units can use data generated from their EHRs to review and analyze these events and share outcomes with unit staff. Units used retrieved data to conduct chart audits to ensure compliance with documentation standards and to more closely examine abnormal EFM strips and subsequent interventions.

Seven units made changes in their EHRs to replace free text data fields with discrete fields that allowed for easier and less time-consuming retrieval of data when creating reports or performing records reviews. Transitioning to a more electronic process for reviews and audits made it also possible for checks to be done more often and by different levels of staff including unit staff, managers, and directors. Many unit staff noted that monitoring processes of care was an important area that was not currently being addressed at their organization and needed improvement. In words of one participant,

“I think that we’ve been focused on safety, … and [on] being able to measure things that we have in place to ensure that we are actually practicing safety. I think that’s the portion that we’ve been missing, just being able to really examine the data. I think … we just didn’t know it. So now that we’ve been following these projects and going through the process, we really know what we need … and are able to work with our IT department so that our system better fits our needs. It protects our patients and makes sure that they’re safe.”

Unit staff reported several ways in which they were planning or using their EHRs to improve their data tracking and reporting methods. Three units were planning upgrades to their EHR systems to add perinatal-specific add-ons with prebuilt reports and ability to link data in the system for easier collection and reporting. A few units used data reporting systems that work in conjunction with their EHRs and have patient safety measures reports built in and updated frequently to meet government and other reporting standards. Two units began discussions with their IT departments to build or modify perinatal-focused dashboards in their EHRs to include safety data for monitoring and to be put on display for all staff to view regularly.

### Challenges of incorporating health IT

Unit staff faced multiple challenges in configuring their IT systems to aid in meeting the goals of SPPC. Unit requests for modifications to EHRs often take a significant amount of time to execute, requiring months of planning and input that extended beyond the SPPC short timeline of program implementation. Many unit staff reported challenges of overburdened IT staff and long queues for getting requested modifications made by their organization’s IT departments; some units overcame this by having dedicated IT staff available to work closely with the unit staff to make these modifications in a timely manner. Others reported challenges in tracking and reporting data including that it was a difficult process to get data out of their EHR systems because most did not have reports built to easily pull the data and instead had to rely on manual collection of the data, which was very time consuming. Other challenges reported included long review and approval processes consisting of several rounds of reviews and committees; difficulties in reaching physician consensus; working with nonclinical IT staff to understand their clinical needs when developing new order sets; difficulties obtaining the correct resources to help units’ extract data to report; and time to optimize the modifications in the system and ensure that they fit into clinical workflows. Some units which were part of larger hospital systems experienced difficulties in getting orders changed or other requests approved because of policies requiring for all changes to be system wide. A number of unit staff shared having difficulties in getting providers to change workflows and to routinely use new standardized processes of care which involved health IT applications. To address this issue, some units incorporated templates within their EHRs to help guide users through the new process and documentation procedures.

Through the course of making changes to their health IT applications, unit staff reported on several plans for the future around integrating the different electronic systems on the unit to address challenges from the lack of integrated information from their EFM systems or from different records within their EHR. One unit described its plans for integration with an update to its EHR that will result in documentation from the mother’s record to flow into the infant’s record for populating and linking data. The upgrade will also create easier workflows and more integration of physician and nurse documentation. Unit staff planning and implementing EHR upgrades noted the importance of being able to provide input in the planning stages and once implementation occurred to provide feedback on how it is going and potential changes that need to be made once it has gone live.

## Discussion

This study describes ways in which L&D units used their EHRs or other health IT applications to apply science safety principles for advancing perinatal patient safety. Although not a main focus or requirement of SPPC, some integration of health IT into the execution of implementing SPPC was reported by 70% of participants. These participants used and modified health IT applications as a means for customizing and implementing their selected perinatal safety strategies. Participants made efforts to use health IT when implementing the program to aid in improvements in standardization, creating independent checks in unit processes, and learning from defects and monitoring care processes for preventing errors on the unit.

Applying safety science principles through use of health IT allows for informed decision-making and more consistent care to be delivered to patients. Unit staff used order sets, checklists, and risk assessments in their EHRs to help guide procedures and processes on the unit in an effort to improve patient safety. This finding was consistent with the findings by Arora et al., Deering, Tobler, and Cypher, and Miller that these types of electronic tools can help to simply and standardize processes while supporting more complete and consistent documentation [4, 5, 10]. Tools built into the EHRs, such as risk assessments, help unit staff be more aware of potential obstetric emergencies that could occur during deliveries such as obstetric hemorrhage and shoulder dystocia and allow care teams to be prepared in these rare occurrences. Other health IT technologies consistent with recommendations in literature were used in safe medication administration efforts including smart pumps and automatic medication dispensing units to help administer medications in an efficient, consistent, and evidenced-based manner while deterring the chance for human error [2, 3, 15]. The frequent use of these types of integrated health IT applications and tools into L&D units’ processes and procedures demonstrate the need to incorporate useful information on electronic tools such as checklists, risk assessments, order sets, and medication specific applications into perinatal safety initiatives.

Standardization of data in EHRs occurred through adoption of agreed-upon language and data definitions such as nationally recognized and recommended NICHD nomenclature and standardized categories for documenting interpretation of EFM strips [9, 10, 16]. Improvements to standardized electronic documentation allowed for easier workflows using EHRs while ensuring that documentation was being completed consistently and accurately across all providers and staff. Using more discrete fields compared to free text data fields to structure data documentation in the EHR assures data is being captured in patient records while reducing staff cognitive burden, consistent with research showing more complete documentation in EHRs [11]. Standardized data in EHRs make it possible for retrieval and reporting of data, which many units struggled with while participating in SPPC. Routinely monitoring processes of care on units helps to encourage a culture of safety through continual discussions of progress demonstrated on the unit, review of patient safety incidents and medical errors, and potential needs for further improvements to be implemented. Programs focused on promoting perinatal safety should include specific education on the standardizing of electronic documentation and tools for electronic reporting and monitoring as they become a more common practice in L&D units but still not easily performed by unit staff.

The multitude of challenges experienced by an extensive number of participating units including obtaining organization approval, working with their organization’s often overloaded IT departments, and having novice users utilizing EHR systems illustrates the importance of the available resources necessary for patient safety improvement plans involving EHRs. As resources are made available to units for these improvements, unit staff will benefit from becoming active participants in the planning and execution of the changes by providing input throughout the planning, executing, and evaluating process to ensure that the changes align with units’ needs and workflows. Stakeholder input from all perspectives including physicians, nurses, midwives, and other users is important to the process of selection, implementation, and evaluation of health IT applications and technology, because they are the end users of the technology, know the culture and workflows of the unit, and drive process changes [2, 10, 16]. Encouraging L&D units who participate in perinatal safety initiatives and programs to designate or include one or more IT staff allocated to work with their team to plan and execute IT changes for achieving program goals may aid in integrating their health IT that best fits the units’ workflows and reduce delays from IT departments.

Interviews also revealed a need for better integration of non-EHR systems on L&D units, an important need also recognized by organizations such as AWHONN [16]. Units lacked integration of EFM and other systems with their EHRs, so data and information were not always documented in an efficient or consistent manner across clinicians and nurses. Enhancements to EHRs and addition of add-on modules may help to create better integration of systems to allow for better data and documentation flow and improved linking of records of mothers and babies for optimal care delivery. Providing L&D units with information on various health IT applications that can be used in conjunction with their EHR to promote perinatal safety allow more options for units to customize perinatal safety strategies to meet their specific goals.

Perinatal safety programs provide L&D units with useful materials, resources, and tools for aiding units to make modifications to L&D unit teams and processes for promoting improved patient safety. As health IT applications become more prevalent in hospitals and provide increased functionality for procedures and quality improvement, information and resources should be incorporated into perinatal safety toolkits to provide guidance on ways health IT can be used to operationalize safety science principles for improving perinatal safety strategies. As shown in our results, although a majority of units were able to use and modify their health IT applications while implementing SPPC, many challenges existed for units suggesting more support and guidance on how to leverage health IT in their units to promote perinatal safety is needed.

A limitation of this study was that the implementation period was only 10 months, and unit staff spent the first several months working on startup and planning activities with little change being made to processes on the unit. This relatively short SPPC time frame limited the number of improvements that could be completed on units, especially when making modifications to EHR systems, due to IT department project queues. This study relied on self-reported information regarding use of health IT from staff on participating L&D units. Results described in this paper are not based on observations of changes that were made on hospital units nor were data collected regarding health IT in any other data reporting activities as part of this program. Also, while this study examined changes made to health IT systems on L&D units with the intent to improve patient safety, no formal assessment was conducted of the potential impact that these health IT changes had on patient safety outcomes.

## Conclusions

This study describes the use of EHRs and other health IT applications by L&D units that participated in a perinatal patient safety initiative to incorporate safety science principles to improve the quality of care and decrease maternal and neonatal adverse events. In planning and making changes to processes and policies to enhance patient safety, units incorporated health IT into their improvement strategies in a variety of ways. These strategies often included modifications to existing EHRs to enhance standardization of processes and data, development of automated checks to aid in decision-making and care delivery, and monitoring care processes for quality improvement. The modifications and refined uses of these technologies played an integral part in the implementation of SPPC and demonstrates the importance of incorporating health IT as a critical component in perinatal patient safety and quality improvement initiatives.

## Abbreviations

AHRQ: Agency for Healthcare Research and Quality
AWHONN: Association of Women’s Health, Obstetrics and Neonatal Nurses
CFIR: Consolidated Framework for Implementation Research
CPOE: Computerized Provider Order Entry
CUSP: Comprehensive Unit-based Safety Program
EFM: Electronic Fetal Monitoring
EHR: Electronic Health Record
IT: Information Technology
L&D: Labor & Delivery
NICHD: National Institute of Child Health and Human Development
QBL: Quantification of Blood Loss
SBAR: Situation, Background, Assessment, Recommendation
SPPC: Safety Program for Perinatal Care
TeamSTEPPS: Team Strategies & Tools to Enhance Performance & Patient Safety
